# Rearing Behavior as Indicator of Spatial Novelty and Memory in Developing Rats

**DOI:** 10.1111/ejn.70162

**Published:** 2025-06-19

**Authors:** Xia Shan, Anuck Sawangjit, Jan Born, Marion Inostroza

**Affiliations:** ^1^ Institute of Medical Psychology and Behavioral Neurobiology University of Tübingen Tübingen Germany; ^2^ German Center for Mental Health (DZPG), Site Tübingen Tübingen Germany; ^3^ Werner Reichert Center for Integrative Neuroscience University of Tübingen Tübingen Germany; ^4^ German Center for Diabetes Research (DZD) Institute for Diabetes Research and Metabolic Diseases of the Helmholtz Center Munich at the University Tübingen (IDM) Tübingen Germany

**Keywords:** developmental trajectory, exploratory behavior, object–place recognition, spatial episodic memory, spatial learning

## Abstract

Among the various forms of exploration, rearing—where rodents stand on their hind legs—reflects the animal's processing of spatial information and response to environmental novelty. Here, we investigated the developmental trajectory of rearing in response to spatial novelty in a standard object–place recognition (OPR) task, with the OPR retrieval phase allowing for a direct comparison of measures of rearing, object exploration, and locomotion as indicators of spatial novelty and memory. Groups of male rats were tested on postnatal day (PD) 25, PD31, PD38, PD48, and at adulthood (PD84). The OPR task comprised a 5‐min encoding phase with the rat exposed to an arena with two identical objects and, 3 h later, a 5‐min retrieval phase in the same arena with one object being displaced to another arena zone. Rearing increased in response to spatial novelty (i.e., the displaced object) at retrieval relative to encoding, with this increase occurring first on PD31, and thus later than preferential object exploration‐based responses emerging already on PD25. Importantly, zone‐specific analyses during retrieval revealed an increase in rearing events in the (now empty) zone where the displaced object is used to be at encoding. This increase was only observed in adult rats (PD84) and likely indicates the presence of specific object–place associations in memory. These findings evidence rearing as behavior covering aspects of spatial novelty complementary to those of object exploration, thereby enabling a more comprehensive characterization of the emergence of spatial episodic memory during early life.

## Graphical abstract

Rearing behavior is an indicator of spatial novelty and memory in developing rats. In an object–place recognition task, rearing increases in response to the spatial novelty (at retrieval) from postnatal day (PD) 31 onwards, that is, with a different trajectory than object exploration‐based responses (sniffing). Only adult rats (PD84) additionally exhibited rearing in the zone that was formerly occupied by an object, with this zone‐specific rearing likely reflecting the specific object–place association in memory.

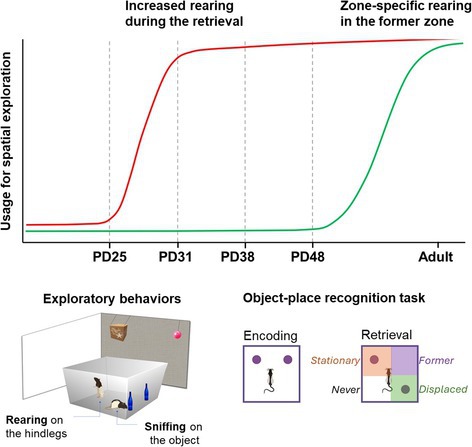

**Abbreviations**
ODI: object discrimination index
OPR: object–place recognition
PD: postnatal day

## 1 Introduction

Spatial exploration is a behavior critical for acquiring novel information about the environment and is used in animals and (nonverbal) human infants to assess the emergence of spatial representations in memory. It manifests in rodents in various forms, including locomotion, object interaction, and rearing, which refers to the animal standing on its hind legs (Eilam and Golani 1989; Lever et al. 2006; Mun et al. 2015; Poulter et al. 2018). Early studies have highlighted rearing as an important exploratory behavior, particularly in novel environments (Hughes 1968; Eilam and Golani 1989; Lever et al. 2006). By increasing height from the ground, rearing is thought to facilitate the processing of distal environmental cues (e.g., visual, olfactory), which are particularly important for orienting directional systems and spatial mapping (Lever et al. 2006; Poulter et al. 2018). Rearing enhances sensory sampling by increasing the visual and olfactory fields, thereby facilitating the processing of these cues (Lever et al. 2006). Recent research underscores the importance of rearing in the context of spatial memory formation (Layfield et al. 2023, Sawangjit et al. 2022). During early development, rearing is critical for forming spatial representations in preweanling rats (Shan et al. 2023).

The control of exploratory rearing likely involves the hippocampus (Lever et al. 2006), a brain region essential to spatial memory and the formation of allocentric spatial representations. Hippocampal theta activity distinctly increases during rearing epochs (Barth et al. 2018), and the frequency of this theta rhythm differentiates whether rearing occurs in a novel or familiar environment (Wells et al. 2013). Inactivating the hippocampus during rearing impairs spatial memory (Layfield et al. 2023). Rearing may specifically support the hippocampal formation of allocentric spatial representation by facilitating the integration of distal cues. These cues enable allocentric navigation during task performance and thereby help in forming cognitive maps and precise spatial representations (Epstein et al. 2017; Tolman 1948).

The object–place recognition (OPR) task is a well‐established method for assessing the formation of spatial allocentric memory in rodents (Ennaceur and Meliani 1992; Mumby et al. 2002; Ennaceur 2010). During the encoding phase of the task, the animal explores two identical objects placed in an arena with specific proximal and distal cues providing the spatial context. At the later retrieval phase, one of the objects is displaced to another, that is, a novel location, and the enhanced time the animal spends exploring, that is, interacting with, the displaced object, in comparison with the time spent exploring the nondisplaced stationary object, is commonly used as an indicator of an allocentric spatial representation in memory. In developmental studies using the OPR tasks, allocentric spatial representation for short (10 min) retention intervals between encoding and retrieval tests was found to emerge in rats at PD16 (Krüger et al. 2012). However, more persistent representations, maintained for more than 2 h, were revealed only after PD24 (Westbrook et al. 2014; Travaglia et al. 2018) and seemed to be fully established at adolescence (~PD38; Contreras et al. 2019). Notably, a similarly protracted developmental trajectory has been observed for episodic‐like object–place context memory (Ramsaran et al. 2016; Asiminas et al. 2022), supporting the idea that memory for the change in the spatial configuration as assessed with the OPR task represents a centerpiece of episodic memory.

So far, the bulk of studies using OPR tasks has exclusively relied on object exploration measures for the assessment of spatial memory, neglecting explorative rearing. Yet, object exploration alone may not provide a valid picture of spatial memory as it does not fully capture the different aspects of the formed spatial representations. Exploratory rearing is associated with enhanced processing distal context cues and, thereby, is indeed expected to cover additional spatial information, that is, on the relation between the object configuration and distal arena context and in this way may provide a more sensitive measure as to the emergence of spatial representations during early development (Lever et al. 2006). Here, to achieve a more comprehensive assessment of the emergence of spatial memory during early development, we examined rearing behavior in response to spatial novelty as established during the retrieval phase of an OPR task and compared the developmental trajectory of rearing with that obtained for the differential object exploration metrics traditionally used in OPR studies. We find that the rearing response to spatial novelty emerges earlier during development and, in addition, is sensitive to different aspects of the spatial memory representation.

## 2 Materials and Methods

### 2.1 Animals

In total, 78 male Long‐Evans rats were used in two experiments (Experiment 1 and Experiment 2). Data from animals in Experiment 1 were partly from a previously published data set (Contreras et al. 2019). For Experiment 1, animals of the different age groups were derived from a total of 15 litters. Four litters were born in our animal facility, which each litter culled to six pups 1 or 2 days after birth. The remaining pups were sourced from Janvier Labs (Le Genest‐Saint‐Isle, France) and arrived at our facility at least 3 days before any manipulation to allow for acclimatization. Each group of animals in Experiment 1, thus, was derived from two litters of six pups each (except for the PD25 group that derived from three litters). In Experiment 2, the 12 rats derived from 6 litters and were sourced by the same vendor (Janvier, Le Genest‐Saint‐Isle, France). After weaning at PD21, the rats were kept in pairs. The whole animal colony was kept at room temperature (22°C ± 1°C) on a 12/12 h light/dark cycle (lights on at 7:00 h). All procedures were performed in accordance with the European animal protection laws and were approved by the Baden‐Württemberg state authority.

### 2.2 Experimental Design and Procedures

**FIGURE 1:**
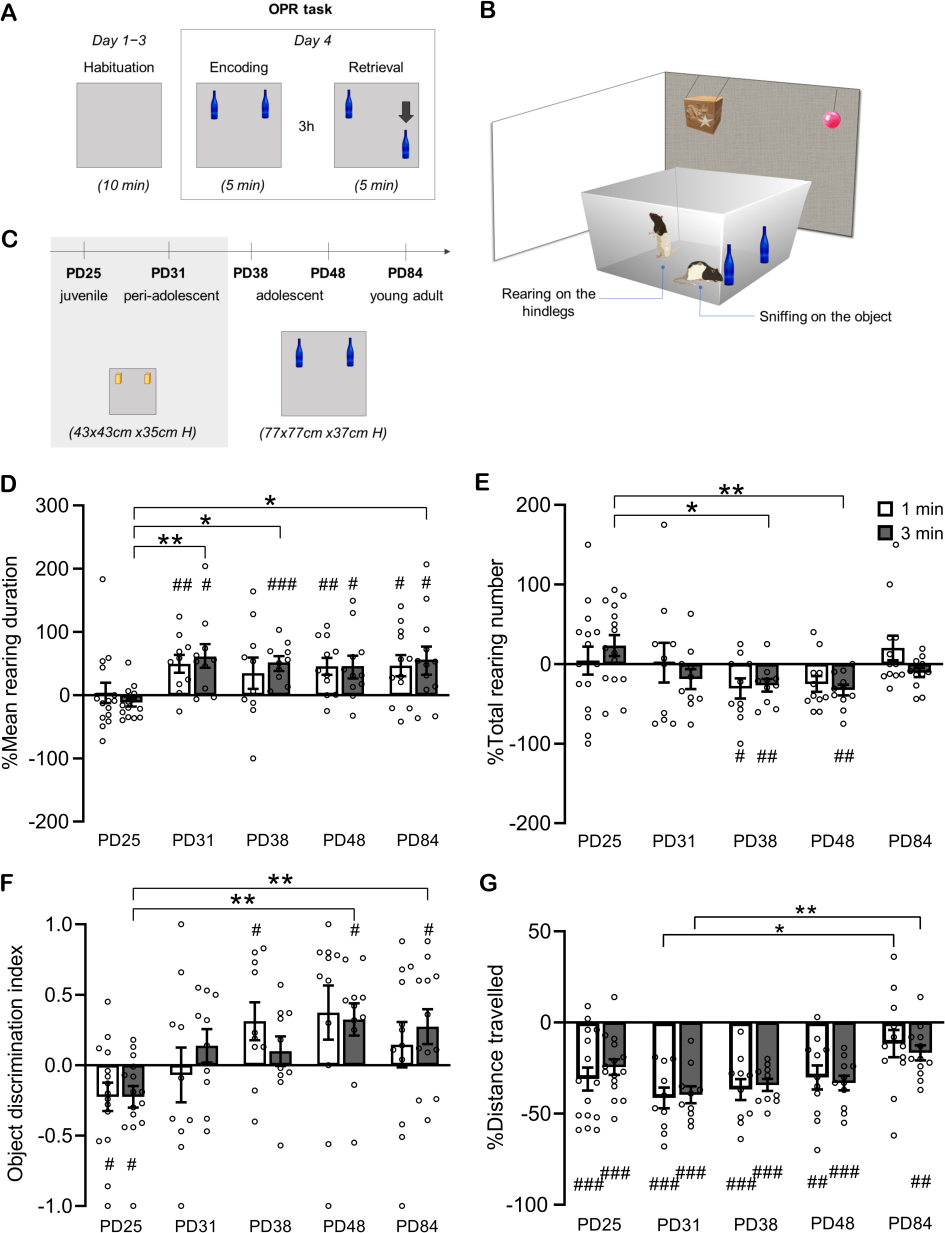
Novelty response to change in object configuration across development. (A) Experimental design and timeline. After 10‐min habituation sessions on the first 3 days, the rats were tested in a standard object–place recognition (OPR) task, comprising a 3‐h retention interval between the 5‐min encoding and 5‐min retrieval phases. During the retrieval phase one of the two (identical) objects placed in the arena during encoding was displaced to a novel location (arrow). (B) Illustration of the open field with distal cues at the surrounding walls, along with an illustration of the rat's exploratory rearing and object exploration behaviors. (C) Rats at different ages (juvenile: PD25, peri‐adolescent: PD31; adolescent: PD38, PD48, and young adult: PD84), were tested in the OPR task with the size of the open field being smaller for the younger (PD25, PD31) than older groups (PD38, PD48, PD84). Based on the encoding of a persistent memory during the encoding phase, the change in the spatial configuration of the two objects is expected to induce a novelty response, which expresses itself in an increase in exploratory rearing and an increased exploration of the displaced object. (D) mean rearing duration (%) and (E) total rearing number (% change from encoding phase), (F) Object discrimination index (as a measure of object exploration), and (G) distance travelled (% change from encoding phase) for each of the five age groups during the first 1‐min (white bars) and 3‐min intervals (gray bars) of the retrieval phase. Mean ± SEM values with overlaid dot plots are shown. #*p* < 0.05, ##*p* < 0.01, ###*p* < 0.001 for one sample *t*‐test against 0. *p < 0.05, **p < 0.01, ***p < 0.001, for Holm–Sidak post hoc tests. Data in F are adapted from Contreras et al. (2019).

Experiments were performed between 7:00 and 14:00 h (i.e., the light phase). In Experiment 1, rats (from different litters) were allocated to the experimental groups based on the postnatal day (PD) on which the OPR task was performed (Figure 1A), i.e., into a juvenile (PD25, *n* = 18), peri‐adolescent (PD31, *n* = 12), adolescent (PD38, *n* = 12; PD48, *n* = 12), and adult (PD84, *n* = 12) group. Before OPR testing, the rats underwent handling for 5 min daily over five consecutive days. For the PD25 group only, handling (PD17‐PD21) was performed in the presence of the mother to mitigate potential stress from nest disturbances. On the following 3 days, all rats underwent a 10‐min habituation session. During habituation, rats freely explored the empty open field, introduced from different sides each session. Afterwards, the rats were habituated, in pairs, to a rest‐box for 6 h. The day after the final habituation session, rats underwent the OPR task. On the task, two identical objects were positioned 9 cm and 15 cm from the respective smaller and larger open field walls. The encoding phase allowed a 5‐min exploration interval. During the subsequent 3‐h retention interval, the rats remained (in pairs) undisturbed in the rest‐box. In the retrieval phase, one of the two objects from the encoding phase was relocated while the other remained in its original position. The retrieval phase allowed the animal to explore the objects for 5 min. Each rat entered the arena from a different side in both encoding and retrieval phases to prevent that the entrance position is used as a proximal cue. Object locations were counterbalanced across rats.

Experiment 2 was a control experiment performed only in adult animals at around PD84 (*n* = 12). The same procedures of handling and habituation as in Experiment 1 were administered. The experiment employed a within‐subject design; that is, on the day following the last habituation session, six rats were tested on the standard OPR task (as described above), and after a 1‐week interval, the same rats performed on a control task (Figure 3A). The other six rats first performed the control task and then the OPR task. The procedures of the control task were the same as those of the OPR task, except that during the retrieval phase, both objects remained at the same location as in the encoding phase, with no displacement.

### 2.3 Apparatus and Objects

In Experiment 1, as described previously (Contreras et al. 2019), the OPR task was performed in a square dark gray open field. Two different sizes of the arena were used depending on the rats' age. The PD25 and PD31 groups performed the task in a smaller arena (43 cm × 43 cm with 35 cm high walls) and the PD38, PD48, and PD84 groups in a larger arena (77 cm × 77 cm with 37 cm high walls). Objects were glass bottles of different shapes, filled with water or sand of different colors. They had sufficient weight to ensure that the rats could not displace them. Two different sets of objects were used depending on the age. For the PD25 and PD31 groups, the height of the objects was 10–18 cm, and for the PD38, PD48, and PD84 groups, it was 22–29 cm. In Experiment 2, we used the same apparatus and objects as used in the PD84 group of Experiment 1. For the OPR and control tasks, different pairs of objects were used in a counterbalanced manner.

To facilitate allocentric navigation during task performance, a number of distal cues were available, and no proximal cues were present inside the arena. The North side of the arena was headed towards a white wall, whereas the East and West sides were surrounded by a gray curtain. The South side of the arena faced a removable black curtain (which was used as entrance for the experimenter). Additional discrete distal cues were provided on the ceiling: a brown wood square (40 cm × 40 cm) located 120 cm above the open field and 36 cm below the ceiling. In the center of the square was a metal square (5 cm × 7 cm) which simultaneously served as a holder for the video camera. At two sides, a pink ball (10 cm diameter) and a light‐brown cartoon box (25 cm × 25 cm × 10 cm), respectively, were attached to the curtains. Two fluorescent strip lights at the side and below the arena provided indirect light. White noise was presented at a constant intensity during all procedures to mask any disturbing sounds. Objects and the arena were cleaned thoroughly after each visit with 70% ethanol solution.

### 2.4 Behavioral Analyses

The rat's behavior was scored offline using the ANY‐Maze tracking software (Stoelting Europe, Dublin, Ireland). Rearing behavior and object exploration were visually scored using Anymaze and calculated for each session, as previously described (Sawangjit et al. 2022). An exploratory rearing event was scored when the rat stood on its hind legs in an upright position, lifting the forelegs off the ground, with or without support from the arena wall or object. Exploratory rearing was only scored when the animal raised its head high and showed signs of scanning the environment, indicative of processing distal cues. Behaviors such as rearing combined with sniffing within 1 cm of an object were not classified as rearing behavior. But rearing was scored if the rat used the object as physical support while looking at distal cues. For both the encoding and retrieval phases, the number of rearing events (total rearing number), the total duration of rearing (total rearing duration [s]), and the mean rearing duration (i.e., the total rearing duration divided by the number of rearing events) were determined. To focus on changes during the retrieval phase, values for the retrieval phase were normalized and expressed as percentage change with values during encoding set to 100%, according to the following formula (here, mean rearing duration): % mean rearing duration at retrieval (%Mean rearing duration) = [(mean rearing duration at retrieval—mean rearing duration at encoding) /mean rearing duration at encoding] * 100. Values for the number of rearing events were transformed in the same way (%Total rearing number). Thus, a positive value indicated an increase in the respective rearing parameter during the retrieval phase with reference to encoding, suggesting that the rats recognized the change in the configuration of the two objects during the retrieval phase, whereas a value close to zero or even negative indicated that rearing was similar in both phases or even diminished in the retrieval phase, suggesting that the rat was not recognizing any change and rather habituated to the arena environment.

Exploration was defined by the rat directing its nose to the object and sniffing. Climbing on an object or sitting next to it without any signs of active exploration was not included. The object discrimination index (ODI) yields meaningful data only if the animal exhibits minimum amounts of exploration of the two objects. Therefore, only rats that had explored each of the two objects for at least 1 s during the encoding phase were included in the analyses. Based on this criterion (and the removal of statistical outliers, see Section 2.5), the final group size for the analyses of the ODI was for PD25 *n* = 15, PD31 *n* = 10, PD38 *n* = 10, PD48 *n* = 11, and PD84 *n* = 12. To assess object exploration, we calculated the ODI which is the standard way to assess OPR memory in adult rats and is defined by the following formula: ODI = [(exploration time for novel object‐location—exploration time for familiar object‐location) / (exploration time for novel object‐location + exploration time for familiar object‐location)]. In addition, the distance travelled was calculated for the encoding and retrieval phases, with the values for the retrieval phase expressed as percentage change values during encoding (in the same way as rearing parameters).

### 2.5 Statistical Analyses

Statistical analyses were performed using SPSS software (IBM, Armonk, NY, USA). Statistical outliers were excluded from the analyses when the respective value exceeded ±2 standard deviations from the group's mean. Analyses of the behavioral measures concentrated on cumulative scores for the first 1 min and the first 3 min of the retrieval phase. The analyses were restricted to the first 3 min of the retrieval phase because this initial interval has been consistently found to most sensitively reflect behavioral memory effects of the task in adult rats whereas exploratory behavior declines thereafter making longer bins, such as 5‐min intervals, less informative (Barker et al. 2007; Dix and Aggleton 1999; Mumby et al. 2002; Oyanedel et al. 2019; Ozawa et al. 2011). Indeed, results of the present study did not essentially change when analyses were based on the total 5‐min retrieval period. As there is, however, some temporal variability in the dynamics of exploration behavior during these first 3 min of the test phase, we examined exploratory behavior for two, that is, the 1‐ and 3‐min cumulative bins, to more precisely capture a potential dependency of this temporal dynamics on the animal's age. To analyze age effects, we used analyses of variance (ANOVAs) with Age as group factor and as repeated measures factors a 1 min/3 min Time bin interval factor (representing the first 1 min and the first 3 min of the retrieval phase). A Zone factor (Former/Never or Same/Novel) is where Former and Never represent the quadrant of the arena where the displaced object had been located at encoding and the quadrant where no object was placed at both the encoding and retrieval phase. And Same and Novel represent the zone where the nondisplaced object was located and the zone where the new location of the displaced object, respectively. Significant ANOVA main or interaction effects were followed by post hoc tests. Specifically, for repeated‐measures ANOVA, the Holm–Sidak post hoc test was used. For other comparisons, Tukey's multiple comparisons test was applied. One‐sample *t*‐tests were used to test whether percentage change values (with reference to the encoding session) significantly differed from zero. Pearson correlation coefficients were calculated between rearing activity: mean rearing duration (%), total rearing number (%), the ODI, and the distance travelled (%), separately for the different age groups, to estimate the interdependency of these measures. Generally, results are reported as means ± SEM. A *p* < 0.05 was considered significant.

## 3 Results

### 3.1 Rearing Increases in Response to the Change in Spatial Configuration First on PD31

In Experiment 1, we examined the age at which rearing increased in response to spatial novelty, that is, the change in the object configuration during the OPR retrieval phase. The percentage change in mean rearing duration at retrieval (with values at encoding set to 100%) differed across age groups, as indicated by a main effect of Age in a 5(Age) × 2(Time bin) repeated measures ANOVA (*F*(4,53) = 3.815, *p* = 0.008). No significant main or interaction effect was observed for the time bin factor (*p* > 0.561; Figure 1D). The juvenile rats on PD25 showed no change in rearing during the OPR retrieval phase in comparison with the encoding phase (*t*(14) = 0.218, *p* = 0.831 and *t*(14) = −1.864, *p* = 0.083, for the first 1 and 3 min of retrieval phase, respectively, one sample *t*‐test against zero). However, the peri‐adolescent rats on PD31 already displayed a significant increase in mean rearing duration for the 1 and 3 min‐interval of the retrieval phase (1 min: *t*(9) = 3.472, *p* = 0.007, 3 min: *t*(9) = 3.099, *p* = 0.013), and this increase was consistently present at all later ages, that is, at PD38 (1 min: *t*(9) = 1.410, *p* = 0.192; 3 min: *t*(9) = 5.351, *p* < 0.001), PD48 (1 min: *t*(9) = 3.283, *p* = 0.009; 3 min: *t*(9) = 2.761, *p* = 0.022) and at PD84 (1 min: *t*(11) = 2.773, *p* = 0.018; 3 min: *t*(11) = 2.731, *p* = 0.020. Figure 1D). The percentage change of mean rearing duration did not differ between PD31, PD38, PD48, and PD84 (Post hoc Holm‐Sidak's test; all *p* > 0.620), indicating that the relative increase in rearing duration at retrieval compared to encoding stabilizes from PD31 onwards (this ratio‐based measure reflects a combination of total duration and frequency. For age‐related changes in raw values, see Section 3.4 and Figure S1‐1). There were also differences across development in the total number of rearing (%) events indicated by a main effect of Age in a 5(Age) × 2(Time bin) repeated measures ANOVA (*F*(4,53) = 2.675, *p* = 0.042, *F*(4,53) = 2.045, *p* = 0.101, for Age × Time bin). Groups PD38 and PD48 showed a transient decrease in the total rearing number (%) during the retrieval phase compared with the encoding phase (PD38 1 min: *t*(9) = −2.406, *p* = 0.039; 3 min *t*(9) = −3.380, *p* = 0.008 and PD48 3 min *t*(9) = −4.302, *p* = 0.002; Figure 1E. See Extended Figure S1‐1 for additional data on rearing duration and rearing number during encoding and retrieval phases of the OPR task).

### 3.2 The ODI Indicates Spatial Novelty First on PD25

Results from preferential object exploration in response to the change in configuration of the two objects during the OPR retrieval phase, that is, the ODI, are summarized in Figure 1F. Like percentage change of mean rearing duration, the ODI also differed across age groups as indicated by a main effect of Age in a 5(Age) × 2(Time bin) repeated measures ANOVA (*F*(4,53) = 4.226, *p* = 0.005). This effect did not depend on whether the first 1 min or 3 min intervals of the retrieval phase were analyzed (*p* > 0.286, for respective main and interaction effects). However, compared to the rearing response, the ODI indicated a distinctly different developmental trajectory. Importantly, the ODI indicated a preferential exploration to the nondisplaced object (i.e., a negative ODI) already in the youngest rats tested on PD25 (1 min: *t*(14) = −2.232, *p* = 0.042, 3 min: *t*(14) = −2.955, *p* = 0.010, one sample *t*‐test against zero, Figure 1F). In the more mature rats, object exploration changed to preferential exploration of the object displaced to the novel location, which resulted in a significant positive ODI reaching the first‐time significance on PD38 (Figure 1F). Total object exploration during encoding did not differ between the age groups, as indicated by one‐way 5 (Age) ANOVA (*F*(4,53) = 1.723, *p* = 0.159, Figure S1‐1C).

We additionally assessed locomotion in terms of distance travelled during the retrieval phase. An increase in locomotion can indicate a rather basic novelty response, as seen, for example, in pups before weaning (Shan et al. 2022). The distance travelled during the OPR retrieval phase, however, did not increase but significantly decreased, relative to values at encoding, in all age groups (Figure 1G), indicating that locomotion was primarily driven by global habituation to the arena environment despite the introduction of the change in object configuration. This decrease was most pronounced at PD31 and then gradually vanished with age, indicated by a main effect of Age (*F*(4,53) = 3.837, *p* = 0.008). However, this effect was not influenced by the Time bin factor, as reflected by nonsignificant main and interaction effects in a 5 (Age) × 2 (Time bin) repeated measures ANOVA (*p* > 0.329).

### 3.3 Correlations Between the Different Behavioral Indicators of Spatial Novelty

We calculated Pearson correlation coefficients between rearing measures (%Mean rearing duration, %Total rearing number), the ODI, and the distance travelled (%Distance travelled) during the OPR task, in order to estimate to what extent the different indicators of spatial novelty may reflect a common underlying process regulating spatial behavior. These exploratory analyses did not reveal any significant (*p* < 0.05, uncorrected for multiple comparison) coefficients between the target measures, that is, %Mean rearing duration, ODI, and %Distance travelled (Figure S5‐1). The only consistently positive correlation appeared to be between the distance travelled (%) and the total rearing number (%) (i.e., a rearing parameter that, in our analyses, was not sensitive to spatial novelty; see Extended Data Table S1, for a summary of correlations). These analyses, thus, are in line with the view that these three measures of spatial novelty indeed reflect separate (i.e., uncorrelated) aspects of spatial behavioral regulation.

### 3.4 Rearing During the Encoding Phase

A final analysis pertained to rearing activity during the 5‐min encoding phase of the OPR. The encoding phase can be considered a condition with readily apparent novelty for animals at all age groups and, thus, may provide a baseline estimate for the maturation of exploratory rearing. Mean rearing duration (s) at encoding phase in the youngest PD25 pups appeared to be slightly longer than in the older pups (*F*(4,53) = 4.386, *p* = 0.004, for effect of Age; one‐way 5 (Age) ANOVA followed by Tukey's post hoc test. See Figure S1‐1 for pairwise comparisons between groups), possibly related to their immature motor skills at that time. By contrast, the total number of rearing and the total rearing duration (s) events showed a rather linear increase with increasing age of the animals (*F*(4,53) = 14.994, *p* < 0.001; *F*(4,53) = 4.242, *p* < 0.005, one‐way 5(Age) ANOVA followed by Tukey's post hoc test, respectively), overall suggesting a rather protracted development of exploratory rearing extending well into early adulthood.

### 3.5 Rearing to Previously Occupied Object Location Emerges Not Before PD84

**FIGURE 2:**
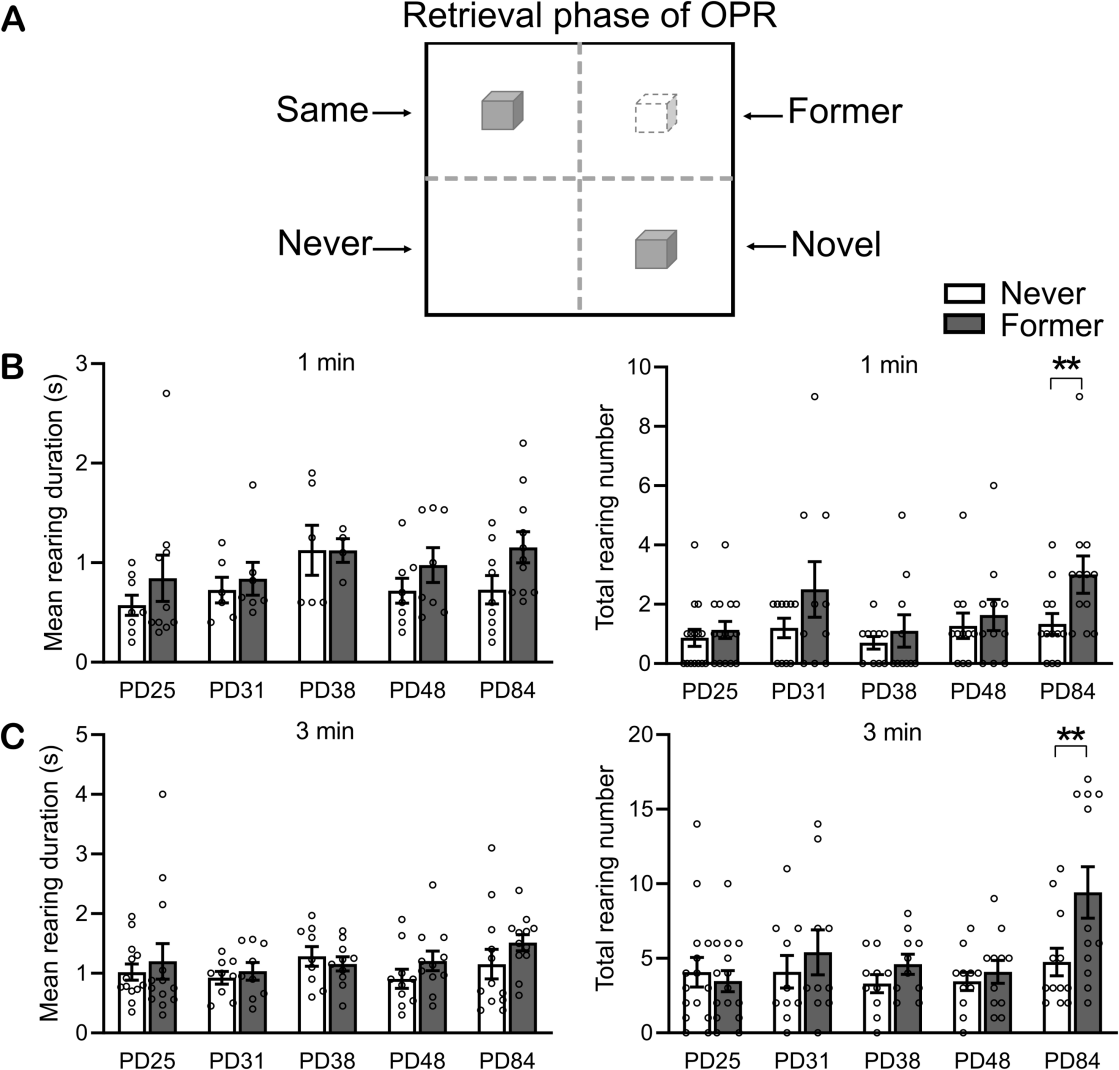
Rearing activity in the Never vs Former zones of the arena. (A) For the analyses, the arena as used during the retrieval phase was divided into 4 quadrants (zones): The Same zone was defined by containing the nondisplaced object, the Novel zone by containing the displaced object, the Never zone by containing no object during both the encoding and retrieval phases, and the Former zone by having contained the displaced object during the encoding phase and being empty during the retrieval phase. (B) Mean rearing duration (s) and total rearing number in the Former and Never zones in the different age groups (PD25, PD31, PD38, PD48, PD84) for the first 1 min and (C) 3 min of the retrieval phase. Mean ± SEM values with overlaid dot plots are shown. *p < 0.05, **p < 0.01, for paired samples *t*‐test.

For a more detailed analysis of how rearing relates to the spatial representation of the arena at the retrieval phase, we divided the arena into four quadrants (zones), that is, (i) the Same zone (where the nondisplaced object was located), (ii) Novel zone (the new location of the displaced object), (iii) Never zone (where no object was placed during the encoding or retrieval phase), and (iv) the Former zone (the now empty zone where the displaced object had been located at encoding, Figure 2A). Our focus was on the two zones without an object, that is, the Never and Former zones, to determine whether rearing discriminates between locations that were or were not occupied by an object at encoding. Comparing rearing in the Former zone with the Never zone, moreover, ensured that, here, rearing reflected a response to spatial memory and novelty, rather than interactions driven by the presence of objects. As the functional meaning of the zones changed from the encoding to the retrieval phase, the analyses relied on absolute values (during the retrieval phase), rather than percent change values (from encoding phase). Whereas mean rearing duration (s) remained unchanged in these analyses (all *p* > 0.071), analyses of the total number of rearing events revealed quite robust effects of age, somewhat more pronounced for the first three than 1 min interval (a 5 (Age) × 2 (zone) × 2 (Time bin) repeated measures ANOVA; *F*(4,53) = 3.664, *p* = 0.010, *F*(4,53) = 4.101, *p* = 0.006, for Age main effect and Age × 1 min/3 min interaction, respectively). Importantly, focusing the analyses on rearing events in the Former and Never zones in the different age groups revealed comparable rearing event numbers for the two zones in all age groups, except in the adult rats, that is, PD84. In the adult rats, the total number of rearing events was consistently higher in the Former than Never zone for both time intervals (1 min: *p* = 0.008, 3 min: *p* = 0.003, in all younger groups: *p* > 0.056, pairwise comparison, Figure 2B,C). We also performed analyses of rearing behavior for the Same and Novel zones which were of an exploratory kind because the object located in these zones is expected to contaminate rearing activity. Indeed, variability of mean rearing duration (s) and total number of rearing events in these zones appeared to be higher than in the other two zones and did not differ in any of the age groups examined (*p* > 0.084 for all relevant group age comparisons, Figure S2‐1).

### 3.6 Increased Rearing During OPR Retrieval Is Caused by the Change in Object Configuration

**FIGURE 3:**
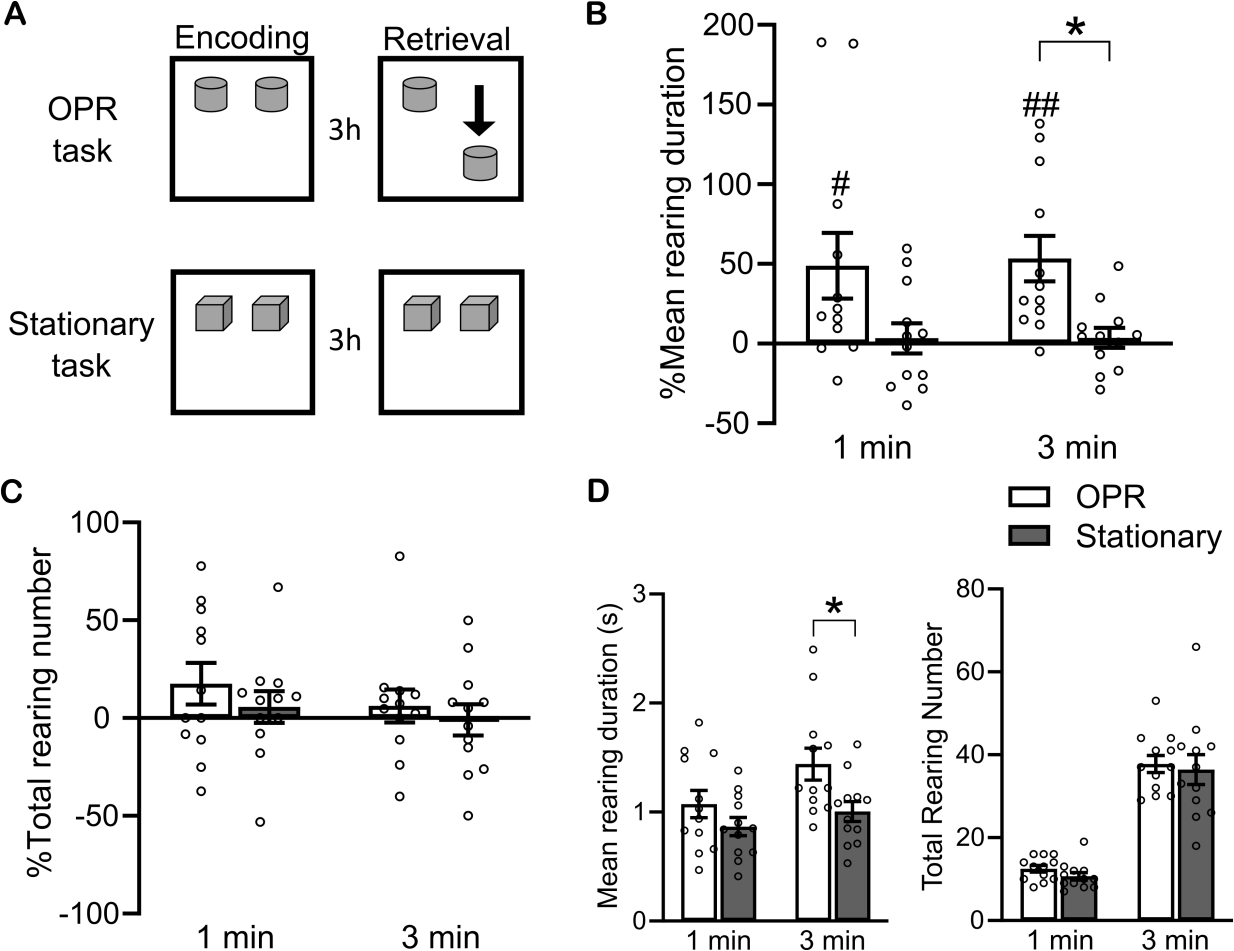
Rearing duration increases in response to change in the object configuration at OPR retrieval testing. (A) Experimental procedure: Following a within‐subject comparison, adult rats were tested in the standard OPR task condition (the same as in Experiment 1, white bars) and in a “stationary” task control condition (gray bars) where the two objects remained at the same location during the Encoding and Retrieval phases. (B) Mean rearing duration (%) and (C) total rearing number (%) during the Retrieval phase, as percent change from values during encoding (set to 100%). (D) Mean rearing duration (s, left) and total rearing number (right) as absolute values during the retrieval phase. Mean ± SEM values with overlaid dot plots are shown for the first 1 min and 3 min of the Retrieval phase. # *p* < *p* < 0.05, ## *p* < 0.01 for one sample *t*‐test against 0. *p < 0.05 for paired samples *t*‐test.

Exploratory rearing is typically oriented towards distal environmental stimuli and not necessarily towards the object of the arena. Against this backdrop, it could be argued that the increase in % mean rearing duration in the retrieval phase we observed in our rats from PD31 on (Figure 1D) is a nonspecific increase in exploratory behavior that is due to the rats' second visit to an objects‐containing arena, rather than reflecting a response specifically related to the change in the configuration of the two objects introduced for retrieval testing. To address this question, we performed a control experiment (Experiment 2) in adult rats (~ PD84) which, in a within‐subject comparison, were tested once on the same standard OPR task used in the main Experiment 1 and in another condition on a “stationary” control task, where both objects during the retrieval phase remained at the same location as during the encoding phase (Figure 3A). As we considered these experiments a basic control, we restricted testing to adult animals, waiving separate testing in younger animals. Only in the OPR task condition (main effect of Task in a 2(Task) × 2(Time bin) repeated measure ANOVA; *F*(1,11) = 7.373, *p* = 0.020), the rats, like in Experiment 1, showed a significant increase in mean rearing duration (%) during the retrieval phase, in comparison with values during encoding (*t*(11) = 2.376, *p* = 0.037 and *t*(11) = 3.746, *p* = 0.003, for the first 1 min and 3 min of the retrieval phase, respectively; one sample *t*‐test against zero; Figure 3B). In contrast, during the stationary control task, mean rearing duration (%) in the retrieval phase did not change from that during the encoding phase (all *p* > 0.567). Moreover, changes in mean rearing duration (%) significantly differed between the task conditions only for the first 3 min (*p* = 0.099 and *p* = 0.010, pairwise comparisons for the first 1 min and 3 min, respectively, Figure 3B). For the % of total number of rearing events, no systematic changes were found (Figure 3C). Mean rearing duration (s), but not the total number of rearing events, was significantly enhanced during the retrieval phase of the OPR task condition in comparison with the stationary task condition when absolute values (unreferenced to the encoding phase) were analyzed, with the difference reaching significance for the first 3‐min interval of the retrieval phase (Figure 3D).

The distance travelled (%) in the retrieval phase, compared with the encoding phase, decreased in both task conditions, with this decrease being somewhat more distinct in the stationary task condition than in the OPR task condition (main effect of Task in a 2(Task) × 2(Time bin) repeated measure ANOVA; *F*(1,11) = 5.089, *p* = 0.045; *p* = 0.047 and *p* = 0.379, pairwise comparisons for the first 1 and 3 min, respectively. *t*(11) = −1.185, *p* = 0.261 and *t*(11) = −5.082, *p* < 0.001, *t*(11) = −4.564, *p* < 0.001 and *t*(11) = −3.814, *p* < 0.01, for the first 1 min and 3 min of the retrieval phase for OPR and Stationary tasks, respectively; one sample *t*‐test against zero. Figure S3‐1). The decrease being prominent in both conditions is in line with the view that this measure mainly reflects the animal's gross habituation to the arena environment.

### 3.7 Increased Rearing During OPR Task Retrieval in the Zone Previously Occupied by an Object

**FIGURE 4:**
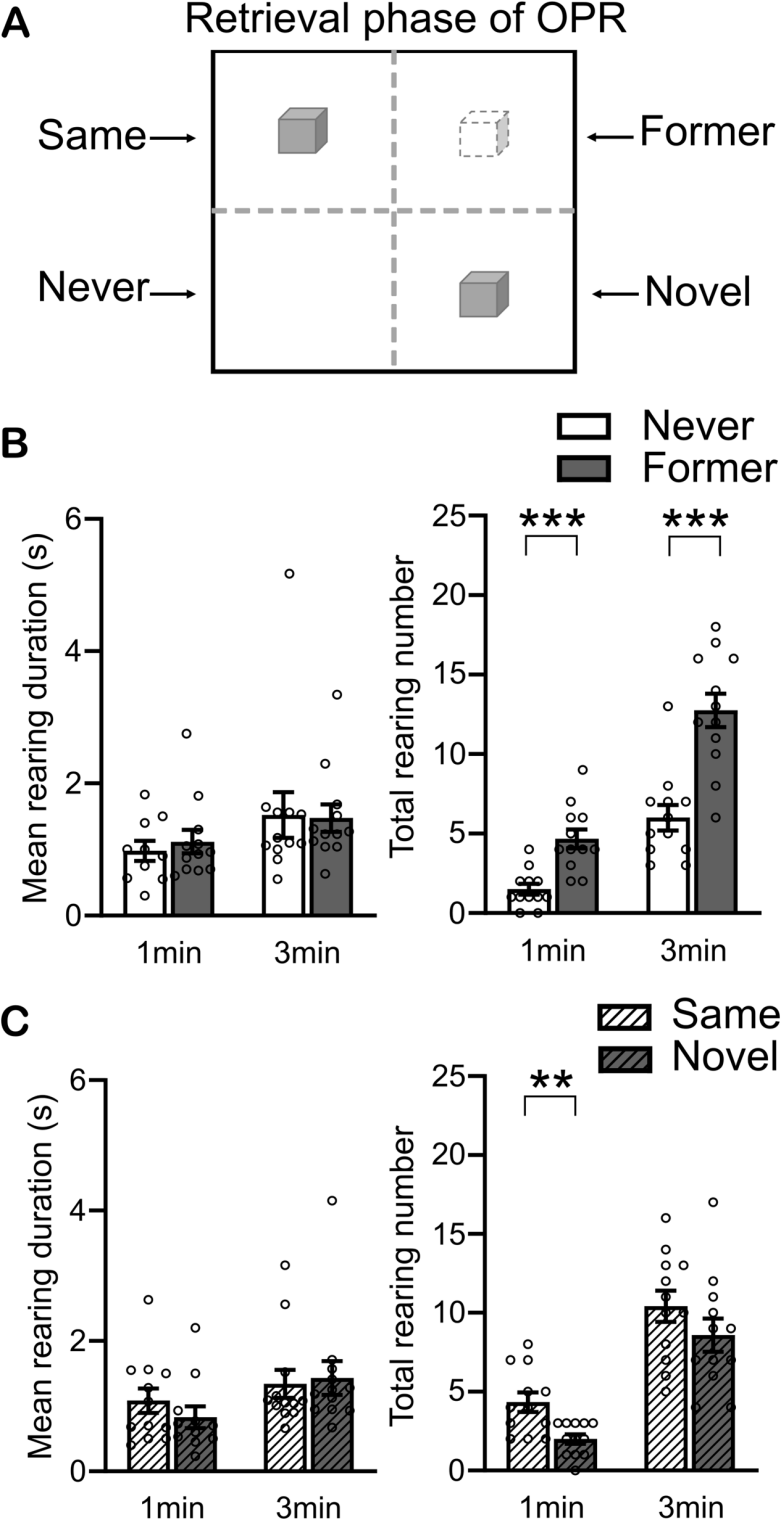
Rearing activity in the four different zones of the arena during the retrieval phase of the OPR task condition. (A) Discrimination of arena zones, see legend to Figure 2A. (B) Mean rearing duration (s, left) and total rearing number (right) in the Former and Never zones for the first 1 min and 3 min of the retrieval phase. (C) The same as in B, for the Same and Novel zones. Mean ± SEM values with overlaid dot plots are shown. *p < 0.05, **p < 0.01, ***p < 0.001 for paired samples *t*‐test.

Like in Experiment 1, we analyzed the dependency of rearing on the arena zone during the retrieval phase of the OPR task in comparison with the stationary task condition (Figure 4A). On the OPR task, rearing during the retrieval phase was increased in the Former zone (where the displaced object used to be during encoding) in comparison with the Never zone. Like in Experiment 1, this increase was present for the total number of rearing events (*p* < 0.001 and *p* < 0.001, for first 1 min and 3 min, respectively, pairwise comparisons, Figure 4B right) but not for the mean rearing duration (s) (*p* = 0.308 and *p* = 0.907, respectively). The exploratory comparison of rearing between the Novel and Same zone revealed a transient (only first 1 min) decrease of the total number of rearing events in the Novel as compared with the Same zone, possibly reflecting a competitive interaction of rearing with object‐related exploration (of the displaced object) which is enhanced in this zone (Figure 4C). Rearing activity during the retrieval phase did not differ between zones that were occupied by an object or empty on the stationary task (Figure S4‐1).

## 4 Discussion

This study in rats assessed the developmental course of rearing in response to spatial novelty as invoked by the change in the object configuration in the retrieval phase of a classical OPR task. We observed an increase in mean rearing duration (%) to spatial novelty beginning on PD31 (peri‐adolescent) which was persistent across subsequent developmental stages, i.e., on PD38, PD48, and during adulthood (PD84). Moreover, the total number of rearing events at the OPR retrieval phase was selectively increased in the now‐empty (“Former”) zone of the arena that had contained the displaced object in the encoding phase of the OPR task, with this zone‐dependent increase emerging only in the adult rats (PD84). A control experiment in adult rats, comparing the classical OPR task with a stationary version where the object locations remained unchanged during the retrieval phase, confirmed that both the increase in mean rearing duration (%) and the selective increase in total rearing event numbers represent specific responses to the change in the object configuration, as experienced during the classical OPR retrieval phase. These findings identify rearing as a behavioral indicator that sensitively reflects different aspects of spatial novelty and memory, emerging at distinct developmental stages (Hughes 1968; Lever et al. 2006; Shan et al. 2023).

Mean rearing duration (%) during the OPR retrieval phase first increased in peri‐adolescent rats (i.e., PD31), which indicates that at this age, the rats are able to recognize the change in the configuration of the two objects and is consistent with many previous studies using different task paradigms and behavioral indicators to show that rats can form allocentric spatial representations allowing them to recognize spatial configurational changes even earlier (Contreras et al. 2019; Shan et al. 2022). Thus, hints that spatial novelty is recognized during the retrieval phase of an OPR task have been obtained in rat pups already at PD16 (Krüger et al. 2012). However, those experiments used only short (< 10 min) retention intervals between encoding and retrieval phases, and more persistent representations maintained over longer (> 2 h) intervals, similar to the present 3‐h interval, were revealed only after PD24 (Travaglia et al. 2018; Westbrook et al. 2014; Krüger et al. 2012). We did not include younger pups in the present study because in foregoing experiments, pups on PD18 did not exhibit sufficient rearing during the 5‐min OPR phases for a reliable assessment of the behavior, although they were basically capable of rearing (Contreras et al. 2019; see also Shan et al. 2023).

Interestingly, in addition to the general increase in rearing activity beginning on PD31, we found a zone‐specific increase in rearing activity selectively in the “Former” zone, that is, the zone of the arena that was empty during the OPR retrieval phase but had contained an object during the encoding phase, with this zone‐specific rearing emerging not until early adulthood (PD84). Apart from its very late emergence, this zone‐specific increase in rearing expressed itself primarily in an increase in the total number of rearing events, rather than in an increase in mean rearing duration. In combination, these differences suggest that the two increases, that is, the general increase in mean rearing duration (%) (on PD31) and the zone‐specific increase in the total number of rearing events (on PD84), reflect different aspects of spatial representation in memory. Whereas the general increase in mean rearing duration (%) on PD31 can be considered to indicate that the rat has recognized some change in the spatial configuration of the two objects, the increase in rearing numbers in the Former zone seems to reflect more specifically the rat's ability to recognize that a previously occupied location is now (at the OPR retrieval phase) empty, that is, it seems to reflect a specific object–place association in memory.

Our finding regarding the zone‐specific increase in rearing, at a first glance, appears to be at variance with a report by Moses et al. (2002) who did not observe an increase in exploratory rearing in adult rats when a familiar object was removed, creating a new empty space. However, that study used four (rather than two) familiar objects and also did not examine zone‐specific effects. On the other hand, the rather late occurrence of the zone‐specific increase in rearing events as an indicator of memory for a specific object–place association remarkably well agrees with findings in rats by Asiminas et al. (2022) indicating the emergence of object–place associations in late adolescence, that is, around 7 weeks of age, while memory for objects was present already at 3–4 weeks of age. That study used a different task approach with the retrieval period involving a change in one of two different objects (instead of a change in the place of one of two identical objects, used in the present study), and it also used a much shorter 2‐min retention interval. The ability to remember and respond to object–place associations relies on complex integrative processes within the hippocampus and interactions with related brain structures, most importantly, the lateral entorhinal cortex and the medial prefrontal cortex (Langston and Wood 2009; Wilson et al. 2013; Chao et al. 2016; Vandrey et al. 2020). Thus, the late emergence of object–place representations for regulating exploratory behavior might be owed to the fact that this system—as core of the episodic memory system—shows a rather slow functional and morphological maturation, in rats as well as in humans (Guillery‐Girard et al. 2013; Caballero et al. 2016; Riggins et al. 2020; Chini and Hanganu‐Opatz 2021). For example, local inhibitory networks within the medial prefrontal cortex that allow gating of hippocampal inputs and thereby regulate spatial exploration do not develop fully until the seventh postnatal week in rats (Tseng and O'Donnell 2007; Caballero et al. 2016; Caballero and Tseng 2016).

Central to our study is the use of a standard OPR retrieval phase to examine the behavioral response to spatial novelty, which represents a quasi‐wholistic approach that, on one side, allows to dissect the contributions of objects and space to the animal's exploration of the environment. On the other side, it allows to compare the different aspects of the animal's exploration behavior. Specifically, it allows to compare, in one and the same environment, the parameters that have been most often used to characterize the development of spatial memory and the response to spatial novelty, which, in addition to rearing, are object exploration, as assessed by the ODI, and locomotion, as assessed by the distance traveled. Notably, we revealed for all of these measures different developmental trajectories, and moreover, in exploratory analyses, these different parameters were not correlated, suggesting that they each reflect different aspects of the animal's representation of the environment and how it is used for exploration. However, it would be premature to exclude any interrelationship between the different measures of novelty detection based on these correlational analyses, as they were derived from rather small sample sizes for each of the age groups. Compared with the mean rearing duration, the object exploration‐based ODI revealed a distinctly earlier emergence of the response to spatial novelty, that is, already at PD25, although at that age exploration preference is for the familiar rather than displaced object (Contreras et al. 2019). This early emergence of the object exploration‐based response matches with the earlier occurrence of object memory (Cruz‐Sanchez et al. 2021), as observed, for example, in developmental comparisons of novel‐object vs. OPR memory (Westbrook et al. 2014) and might also be related to a stronger tendency to regulate spatial behavior based on proximal cues in very young animals, whereas distal spatial cues are integrated into behavioral regulation only later during development (Akers and Hamilton 2007; Ramsaran et al. 2016; Shan et al. 2022).

An increase in locomotion has been found to robustly reflect the recognition of spatial novelty in rat pups on PD16 (Shan et al. 2022). By contrast, in the present study, all juvenile rat groups—between PD25 and PD49—as well as the adult group (PD84) consistently decreased the distance travelled during the OPR retrieval phase, in comparison with the encoding phase, pointing to a predominant habituation towards the arena environment at the second phase visit. Indeed, the arena environment at the second visit altogether contained more familiar than novel aspects, explaining that a rather nonspecific parameter of locomotion, like the distance travelled in the arena, is shifted towards a habituated response. In combination with other studies (Moses et al. 2002; Save et al. 1992; Lever et al. 2006), the findings support the conclusion that in the age range tested here, the change in the object configuration at the OPR retrieval phase represents an insufficient amount of novelty to trigger a nonspecific increase in locomotion. In addition, this parameter is probably confounded by a spontaneous age‐related decrease in locomotion unrelated to any environmental change.

Limitations to our study arise from the use of only male rats, which aimed to reduce behavioral variability due to alterations in sex‐related hormones in female rats. Given evidence for sex‐related differences in rearing behavior (e.g., Lever et al. 2006; Sturman et al. 2018; Layfield et al. 2023), the generalizability of our findings to females requires further testing. Also, in Experiment 1, following animal welfare consideration, we relied on a reduced number of litters for establishing the different age groups, resulting in a diminished genetic background variance. Littermates may not be considered statistically independent observations, raising questions about the extent to which our samples are truly representative (Festing 2006). The use of larger, more diverse samples from independent litters would surely help ensure the generalizability of our findings. Finally, a limitation is also that the “Stationary” control task was only tested in adult rats. Including this control in younger age groups may have helped to further disentangle memory‐related aspects from general exploratory aspects in rearing during development.

**FIGURE 5:**
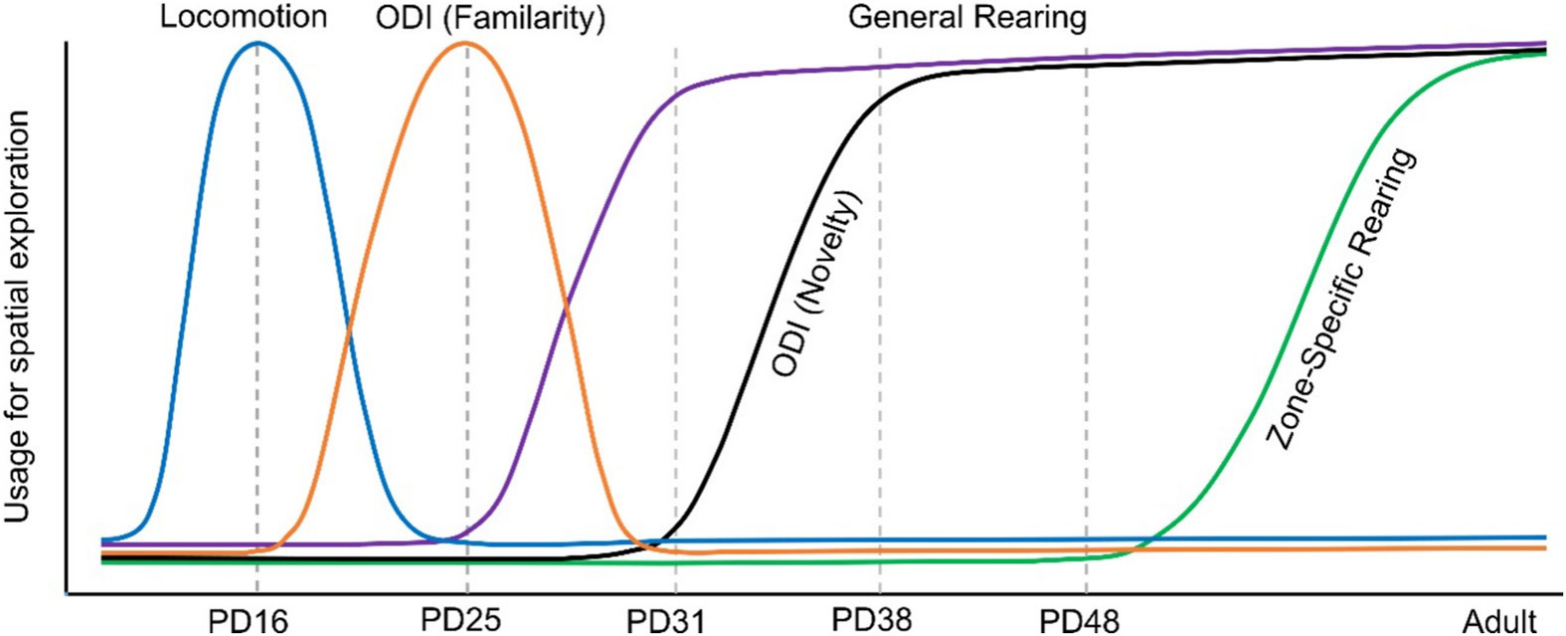
Evolution of spatial exploration behavior during early development. The schema illustrates the animal's dominant response to spatial novelty at different developmental stages, that is, with a nonspecific increase in general locomotion around PD16 (blue line, Shan et al. 2022) with an increase in the object discrimination index (ODI) towards familiar object–locations around PD25 (orange line), with a general and persisting increase in rearing at PD31 (purple), with an increase in the ODI towards novel object locations around PD38 (black), and with a zone specific increase in rearing at previously occupied locations (as an indicator of object–location memory) only after PD48 (green).

In sum, the different behavioral responses to spatial novelty display different developmental trajectories partly owed to the fact that they pertain to different aspects of the animal's environment and its brain representations (Figure 5). Nonspecific increases in locomotor activity as a response to generally perceived novelty vs. familiarity in the test arena are primarily observed in very young pups before PD25, that is, the age range examined here. Object exploration‐based responses to spatial novelty occur earlier (PD25) than rearing‐based novelty recognition responses (PD31). Importantly, at their first occurrence, these responses remain unspecific in that they indicate the rat's recognition of any kind of change in the spatial configuration. Zone‐specific increases in the number of rearing, finally, emerge not until early adulthood (PD84) and refer to the presence of specific object–place associations in memory. Of course, the interpretation of our findings is limited as they account for the standard OPR retrieval situation used in the present study, with changes, that is, in the number of objects or the salience of distal cues, likely inducing more or less slight shifts in the actual emergence of respective novelty responses.

The relatively late occurrence of rearing in response to spatial novelty is probably related to the fact that this behavior involves a rather complex system aiming to form associations between distal spatial contextual cues and objects (Lever et al. 2006; Sturman et al. 2018). The encoding of spatial information during rearing is not only associated with increased theta and gamma activity in hippocampal circuitries (Barth et al. 2018; Layfield et al. 2023) but exploratory rearing is also regulated by amygdala circuits and hypothalamic MCH neurons (Concetti et al. 2024; Moses et al. 2002). Given this integrative function, the rearing response to novelty perhaps offers the most valid behavioral indicator for the presence of specific object–place representations in memory that can also be used in more natural study conditions.

## Author Contributions

**Xia Shan:** formal analysis, investigation, methodology, visualization, writing – original draft. **Anuck Sawangjit:** conceptualization, formal analysis, methodology. **Jan Born:** conceptualization, funding acquisition, methodology, project administration, resources, supervision, validation, visualization, writing – review and editing. **Marion Inostroza:** conceptualization, data curation, formal analysis, funding acquisition, project administration, resources, supervision, validation, visualization, writing – review and editing.

## Ethics Statement

All procedures were performed in accordance with the European animal protection laws and were approved by the Baden‐Württemberg state authority.

## Conflicts of Interest

The authors declare no conflicts of interest.

## Peer Review

The peer review history for this article is available at https://www.webofscience.com/api/gateway/wos/peer‐review/10.1111/ejn.70162.

## Supporting information


**Figure S1‐1.** Rearing activity and object exploration during the OPR encoding and retrieval phases. (A) Mean rearing duration (s), (B) total rearing number, and (C) total rearing duration (s) in the different age groups (PD25, PD31, PD38, PD48, PD84) for the 5‐min OPR encoding phase. (D) Mean rearing duration (s), (E) total rearing number and (F) total rearing duration (s) for the 5‐min OPR retrieval phase. (G) total object exploration in the different age groups (PD25, PD31, PD38, PD48, PD84) for the 5‐min OPR encoding phase. Data were analyzed using one‐way ANOVA followed by Tukey's multiple comparisons test. Mean ± SEM values with overlaid dot plots are shown. *p < 0.05, **p < 0.01, ***p < 0.001.

**Figure S2‐1.** Rearing activity in the Same vs Novel zones of the arena. (A) Discrimination of arena zones, see legend to Figure 2A. (B) Mean rearing duration (s) and total rearing number in the Same and Novel zones in the different age groups (PD25, PD31, PD38, PD48, PD84) for the first 1 min and (C) 3 min of the retrieval phase. Mean ± SEM values with overlaid dot plots are shown.

**Figure S3‐1.** Distance travelled (%) at retrieval in OPR and stationary task condition of Experiment 2. The mean ± SEM distance travelled (%) during the first 1 min and 3 min of the retrieval phase is shown in percent change from levels during encoding (set to 100%). ## *p* < 0.01, ### *p* < 0.001, for one sample *t*‐test against 0. *p < 0.05 for LSD post hoc pairwise tests.

**Figure S4‐1.** Rearing activity in the 4 different zones of the arena during the retrieval phase of the stationary task condition. (A) Corresponding to the analysis of the OPR task the quadrants of the arena were here separated into two kinds of (functionally) different Zones containing an object (Same 1, Same 2) or remaining empty (Never 1, Never 2). (B) Mean rearing duration (s, left) and total rearing number (right) in the two Never and (C) the two Same for the first 1 min and 3 min of the retrieval phase. Mean ± SEM values with overlaid dot plots are shown. There were no significant differences between any of the conditions.

**Figure S5‐1.** Scatter plots showing key correlations between behavioral parameters of spatial novelty detection during the retrieval phase of the OPR task. Left—Correlation between object discrimination index (ODI, *y*‐axes) and %Mean rearing duration and (middle) %Distance travelled. To avoid complexity, the %Total rearing number variable is excluded. Right—Correlation between %Mean rearing duration (*y*‐axis) and %Distance travelled. Pearson correlations are shown separately for each age group. *p < 0.05, **p < 0.01 and ***p < 0.001 level (uncorrected).

**Table S1.** Summary of correlations between the %Mean rearing duration, %Total rearing number, object discrimination index (ODI), and %Distance travelled for the first 3‐min period of the OPR test phase. Since Kolmogorov–Smirnov tests did not support nonnormality of any of the distributions, Pearson's correlation coefficients and respective *p*‐values are indicated in bold (uncorrected). Bottom lines indicate mean correlation coefficients across age groups; p‐values, here, refer to one sided *t*‐tests (against zero) to test systematic shifts towards negative or positive correlation coefficients across age groups (mean correlations and *t*‐tests were calculated on Fisher z‐transformed coefficients).

**Data S1.** Supporting Information.

## Data Availability

The data supporting this study's findings are available from the corresponding author upon reasonable request.
