# Supplementary figures and images for: Rearing Behavior as Indicator of Spatial Novelty and Memory in Developing Rats
Source: Eur J Neurosci. 2025 Jun 19;61(12):e70162. doi: 10.1111/ejn.70162 (PMC12179583; doi:10.1111/ejn.70162)

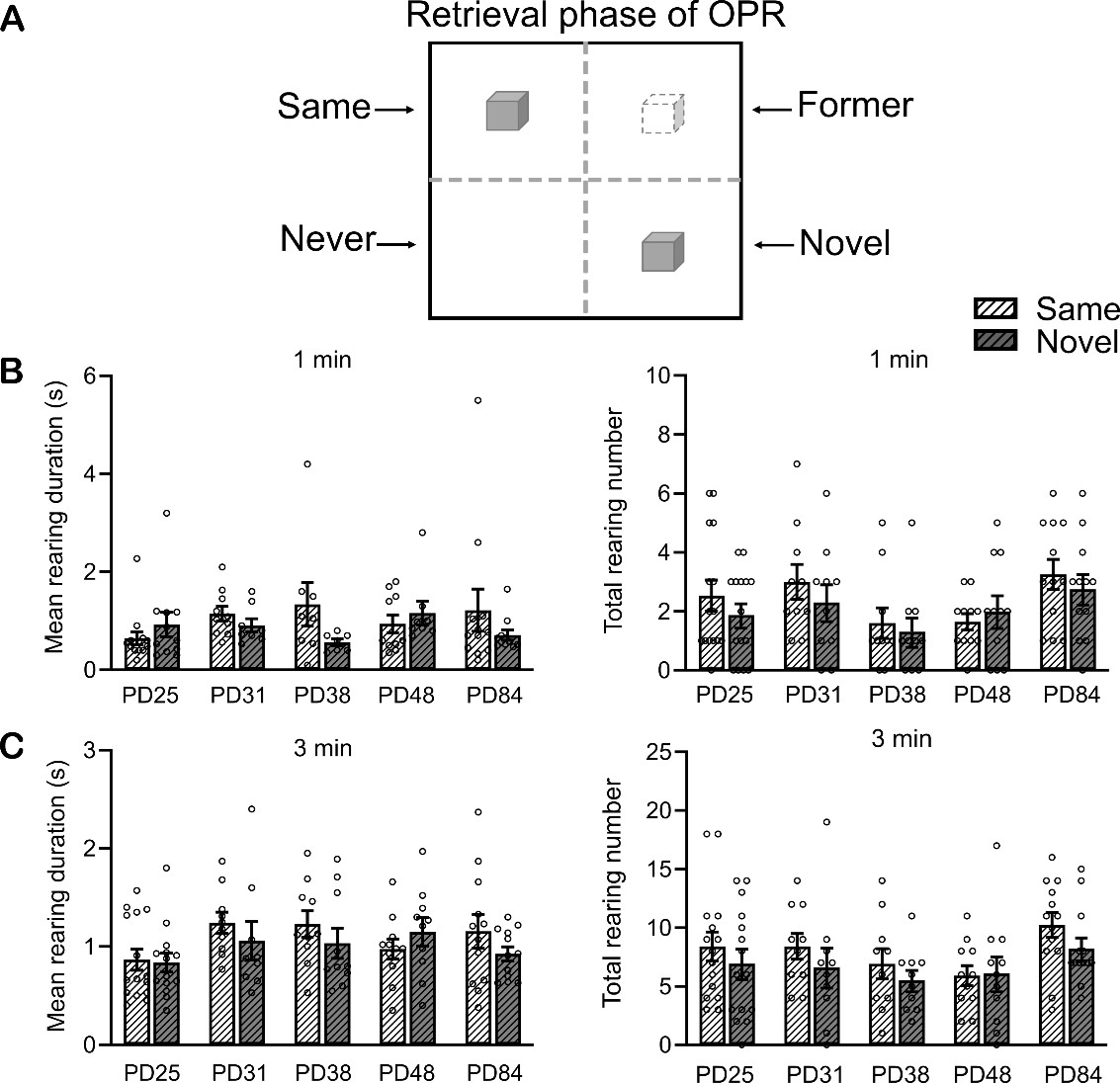

Supplement: Supplementary file 2 — Figure S2‐1. Rearing activity in the Same vs Novel zones of the arena. (A) Discrimination of arena zones, see legend to Figure 2A. (B) Mean rearing duration (s) and total rearing number in the Same and Novel zones in the different age groups (PD25, PD31, PD38, PD48, PD84) for the first 1 min and (C) 3 min of the retrieval phase. Mean ± SEM values with overlaid dot plots are shown. [file EJN-61-0-s001.tiff]

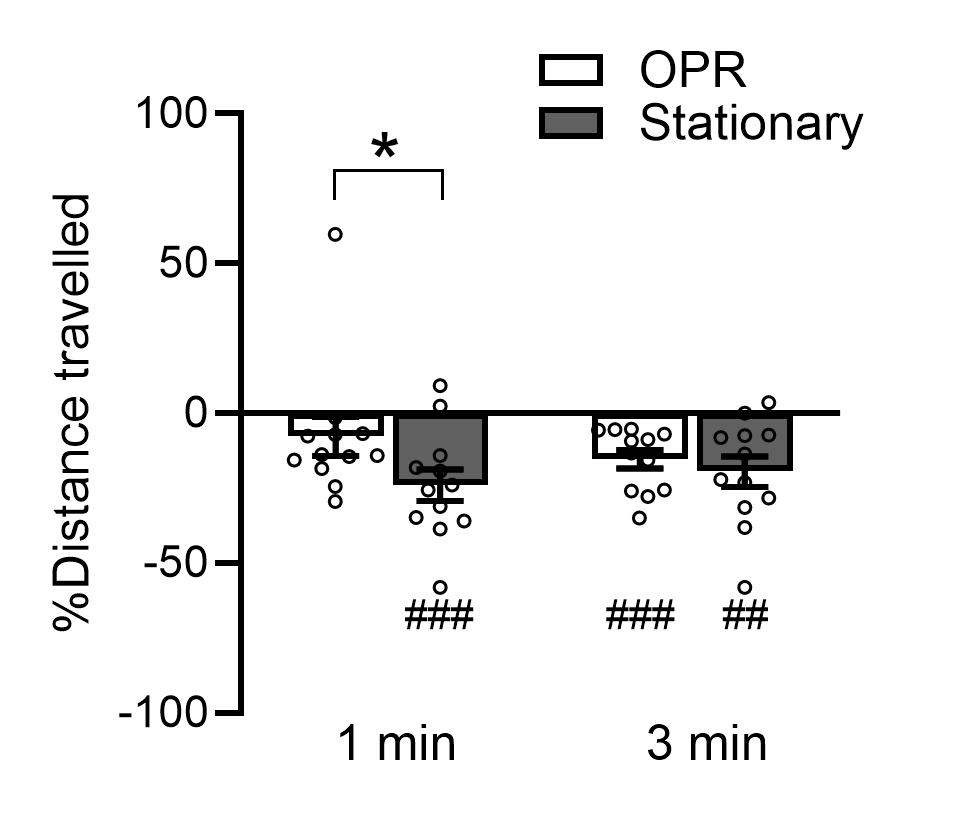

Supplement: Supplementary file 3 — Figure S3‐1. Distance travelled (%) at retrieval in OPR and stationary task condition of Experiment 2. The mean ± SEM distance travelled (%) during the first 1 min and 3 min of the retrieval phase is shown in percent change from levels during encoding (set to 100%). ## p < 0.01, ### p < 0.001, for one sample t‐test against 0. *p < 0.05 for LSD post hoc pairwise tests. [file EJN-61-0-s006.tiff]
