# Supplementary material for: Rearing Behavior as Indicator of Spatial Novelty and Memory in Developing Rats
Source: Eur J Neurosci. 2025 Jun 19;61(12):e70162. doi: 10.1111/ejn.70162 (PMC12179583; doi:10.1111/ejn.70162)
Supplement: Supplementary file 6 — Table S1. Summary of correlations between the %Mean rearing duration, %Total rearing number, object discrimination index (ODI), and %Distance travelled for the first 3‐min period of the OPR test phase. Since Kolmogorov–Smirnov tests did not support nonnormality of any of the distributions, Pearson's correlation coefficients and respective p‐values are indicated in bold (uncorrected). Bottom lines indicate mean correlation coefficients across age groups; p‐values, here, refer to one sided t‐tests (against zero) to test systematic shifts towards negative or positive correlation coefficients across age groups (mean correlations and t‐tests were calculated on Fisher z‐transformed coefficients). [file EJN-61-0-s004.docx]

| **Extended Data Table 1\| Correlations between ODI, exploratory rearing and distance travelled for minute 3 of OPR task** | | | | | | |
| --- | --- | --- | --- | --- | --- | --- |
|  |  |  | | **%Total rearing number** | **ODI** | **%Distance travelled** |
| **%Mean rearing duration** |  | PD 84 | ***r*** | 0.273 | 0.358 | -0.207 |
|  |  |  | ***p*** | 0.390 | 0.253 | 0.518 |
|  |  | PD 48 | ***r*** | -0.104 | 0.263 | -0.233 |
|  |  |  | ***p*** | 0.762 | 0.435 | 0.491 |
|  |  | PD 38 | ***r*** | 0.470 | -0.078 | 0.106 |
|  |  |  | ***p*** | 0.171 | 0.830 | 0.770 |
|  |  | PD 31 | ***r*** | -0.029 | -0.366 | -0.211 |
|  |  |  | ***p*** | 0.936 | 0.298 | 0.559 |
|  |  | PD 25 | ***r*** | -0.229 | 0.171 | -0.106 |
|  |  |  | ***p*** | 0.412 | 0.542 | 0.708 |
|  |  | Average | ***r*** | 0.085 | 0.071 | -0.132 |
|  |  |  | ***p*** | 0.566 | 0.630 | 0.107 |
|  |  |  |  |  |  |  |
|  |  |  |  |  |  |  |
| **%Total rearing number** |  | PD 84 | ***r*** |  | 0.273 | 0.288 |
|  |  |  | ***p*** |  | 0.391 | 0.364 |
|  |  | PD 48 | ***r*** |  | -0.465 | 0.508 |
|  |  |  | ***p*** |  | 0.150 | 0.111 |
|  |  | PD 38 | ***r*** |  | 0.245 | 0.617 |
|  |  |  | ***p*** |  | 0.494 | 0.057 |
|  |  | PD 31 | ***r*** |  | -0.115 | 0.820 |
|  |  |  | ***p*** |  | 0.752 | **0.004** |
|  |  | PD 25 | ***r*** |  | 0.437 | 0.327 |
|  |  |  | ***p*** |  | 0.103 | 0.234 |
|  |  | Average | ***r*** |  | 0.076 | 0.547 |
|  |  |  | ***p*** |  | 0.684 | **0.017** |
|  |  |  |  |  |  |  |
|  |  |  |  |  |  |  |
| **ODI** |  | PD 84 | ***r*** |  |  | 0.054 |
|  |  |  | ***p*** |  |  | 0.868 |
|  |  | PD 48 | ***r*** |  |  | -0.226 |
|  |  |  | ***p*** |  |  | 0.505 |
|  |  | PD 38 | ***r*** |  |  | 0.570 |
|  |  |  | ***p*** |  |  | 0.085 |
|  |  | PD 31 | ***r*** |  |  | 0.250 |
|  |  |  | ***p*** |  |  | 0.486 |
|  |  | PD 25 | ***r*** |  |  | 0.019 |
|  |  |  | ***p*** |  |  | 0.946 |
|  |  | Average | ***r*** |  |  | 0.148 |
|  |  |  | ***p*** |  |  | 0.366 |
