# Supplementary material for: Rearing Behavior as Indicator of Spatial Novelty and Memory in Developing Rats
Source: Eur J Neurosci. 2025 Jun 19;61(12):e70162. doi: 10.1111/ejn.70162 (PMC12179583; doi:10.1111/ejn.70162)
Supplement: Supplementary file 7 — Data S1. Supporting Information. [file EJN-61-0-s002.pdf]

**Rearing behavior as indicator of spatial novelty and memory in developing rats**

Xia Shan[1], Anuck Sawangjit[1,4], Jan Born[1,2,3,4], Marion Inostroza[1]

**Extended Figures**

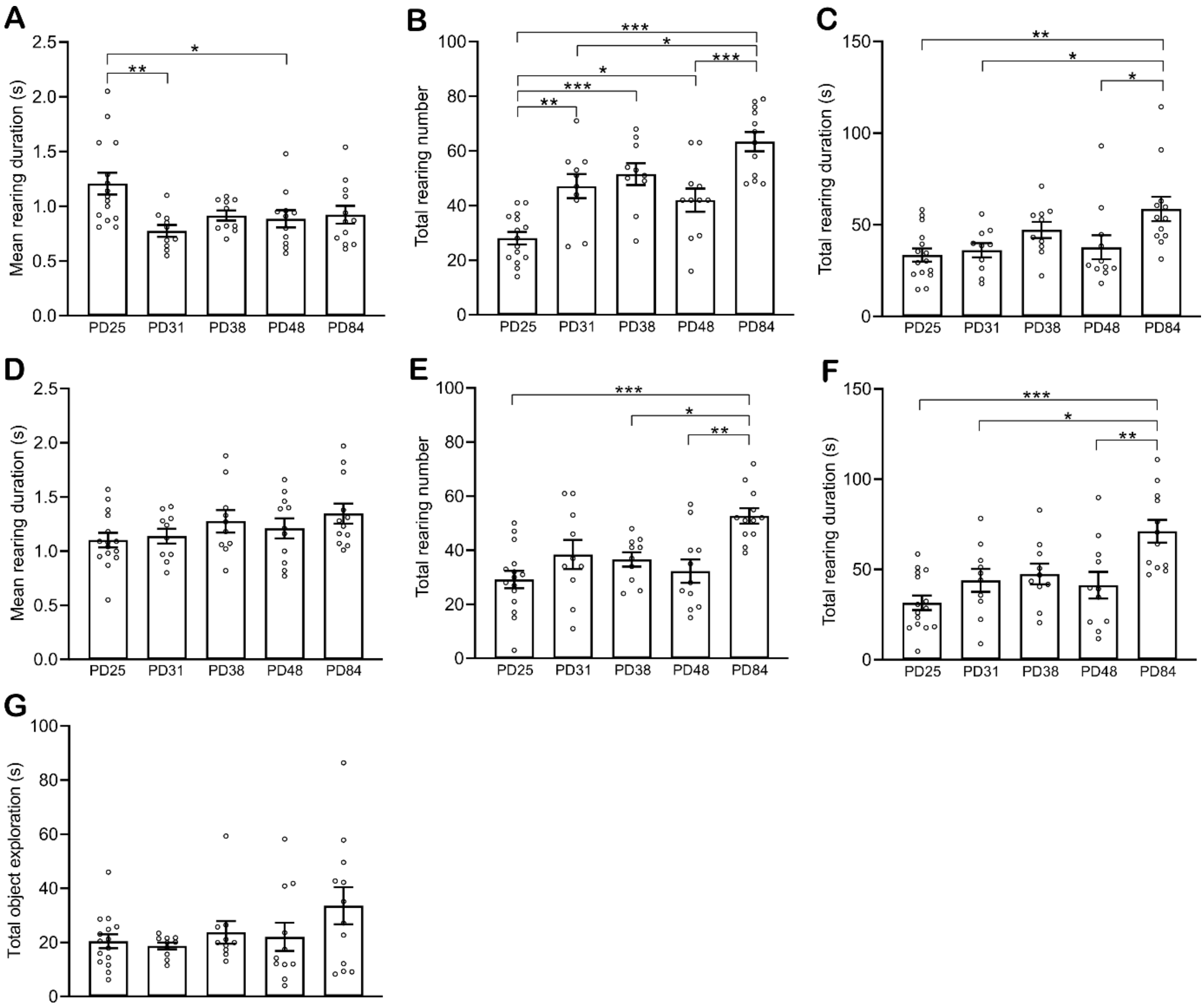


**Figure 1-1**. **Rearing activity and object exploration during the OPR encoding and retrieval phases.** (A) Mean rearing duration (s), (B) total rearing number, and (C) total rearing duration (s) in the different age groups (PD25, PD31, PD38, PD48, PD84) for the 5-min OPR encoding phase. (D) Mean rearing duration (s), (E) total rearing number and (F) total rearing duration (s) for the 5-min OPR retrieval phase. (G) total object exploration in the different age groups (PD25, PD31, PD38, PD48, PD84) for the 5-min OPR encoding phase. Data were analysed using one-way ANOVA followed by Tukey's multiple comparisons test. Mean ± SEM values with overlaid dot plots are shown. *p < 0.05, **p < 0.01, ***p < 0.001.

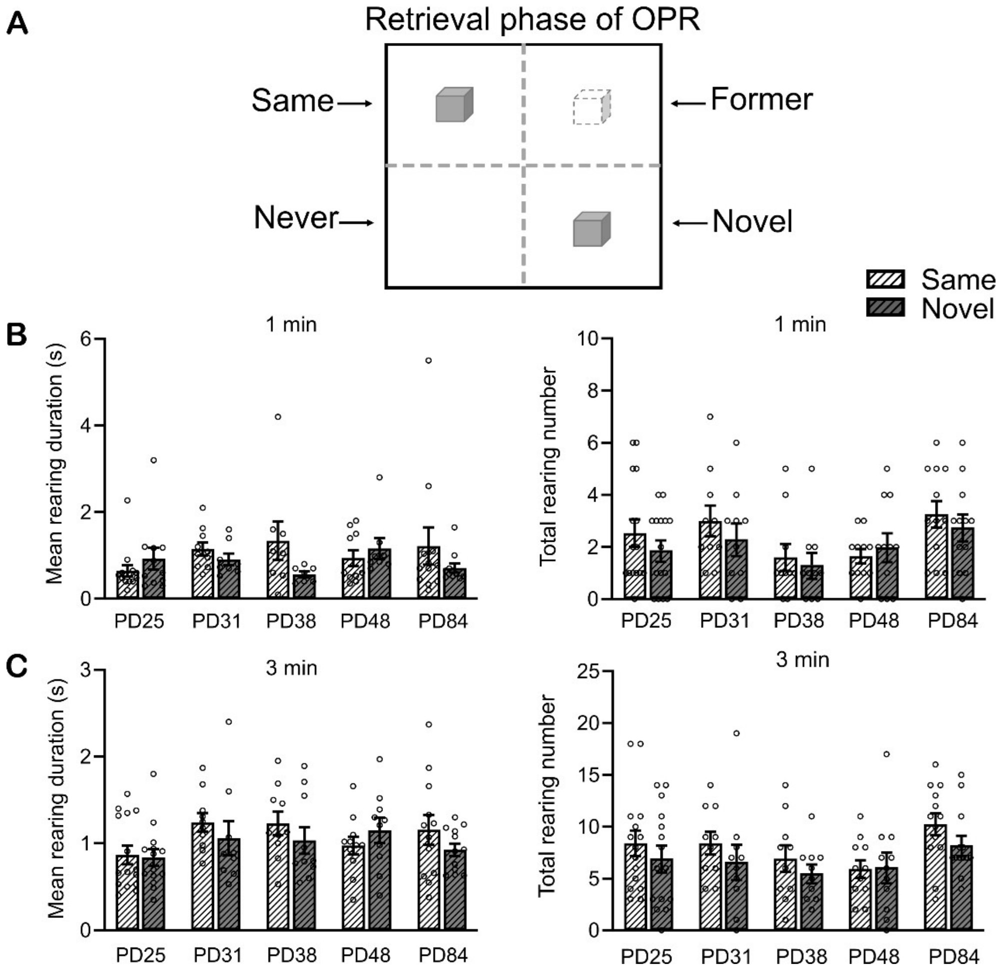


**Figure 2-1. Rearing activity in the Same vs Novel zones of the arena**. (A) Discrimination of arena zones, see legend to Figure 2A. (B) Mean rearing duration (s) and total rearing number in the Same and Novel zones in the different age groups (PD25, PD31, PD38, PD48, PD84) for the first 1 min and (C) 3 min of the retrieval phase. Mean ± SEM values with overlaid dot plots are shown.

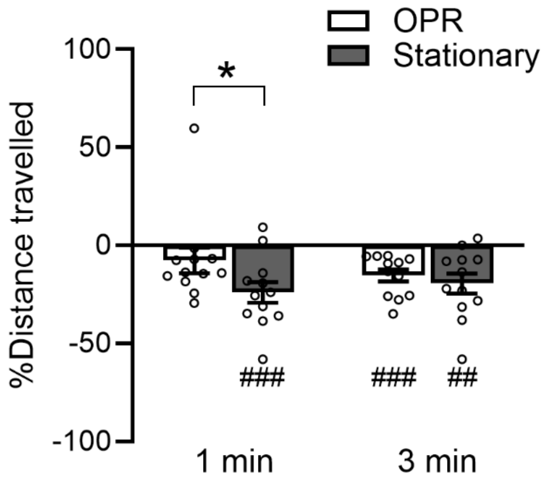


**Figure 3-1**. **Distance travelled (%) at retrieval in OPR and stationary task condition of Experiment 2**. The mean ± SEM distance travelled (%) during the first 1 min and 3 min of the retrieval phase is shown in percent change from levels during encoding (set to 100 %). ## $p < 0.01$, ### $p < 0.001$, for one sample t-test against 0. *$p < 0.05$ for LSD post-hoc pairwise tests.

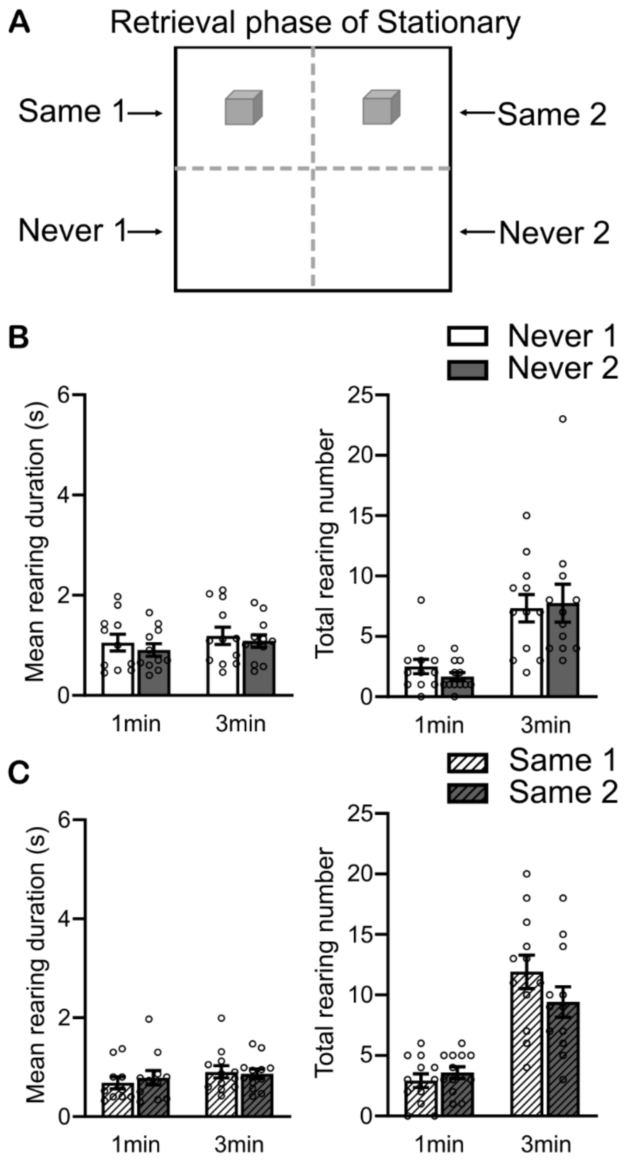


**Figure 4-1**. **Rearing activity in the 4 different zones of the arena during the retrieval phase of the stationary task condition.** (A) Corresponding to the analysis of the OPR task the quadrants of the arena were here separated into two kinds of (functionally) different Zones containing an object (Same 1, Same 2) or remaining empty (Never 1, Never 2). (B) Mean rearing duration (s, left) and total rearing number (right) in the two Never and (C) the two Same for the first 1 min and 3 min of the retrieval phase. Mean ± SEM values with overlaid dot plots are shown. There were no significant differences between any of the conditions.

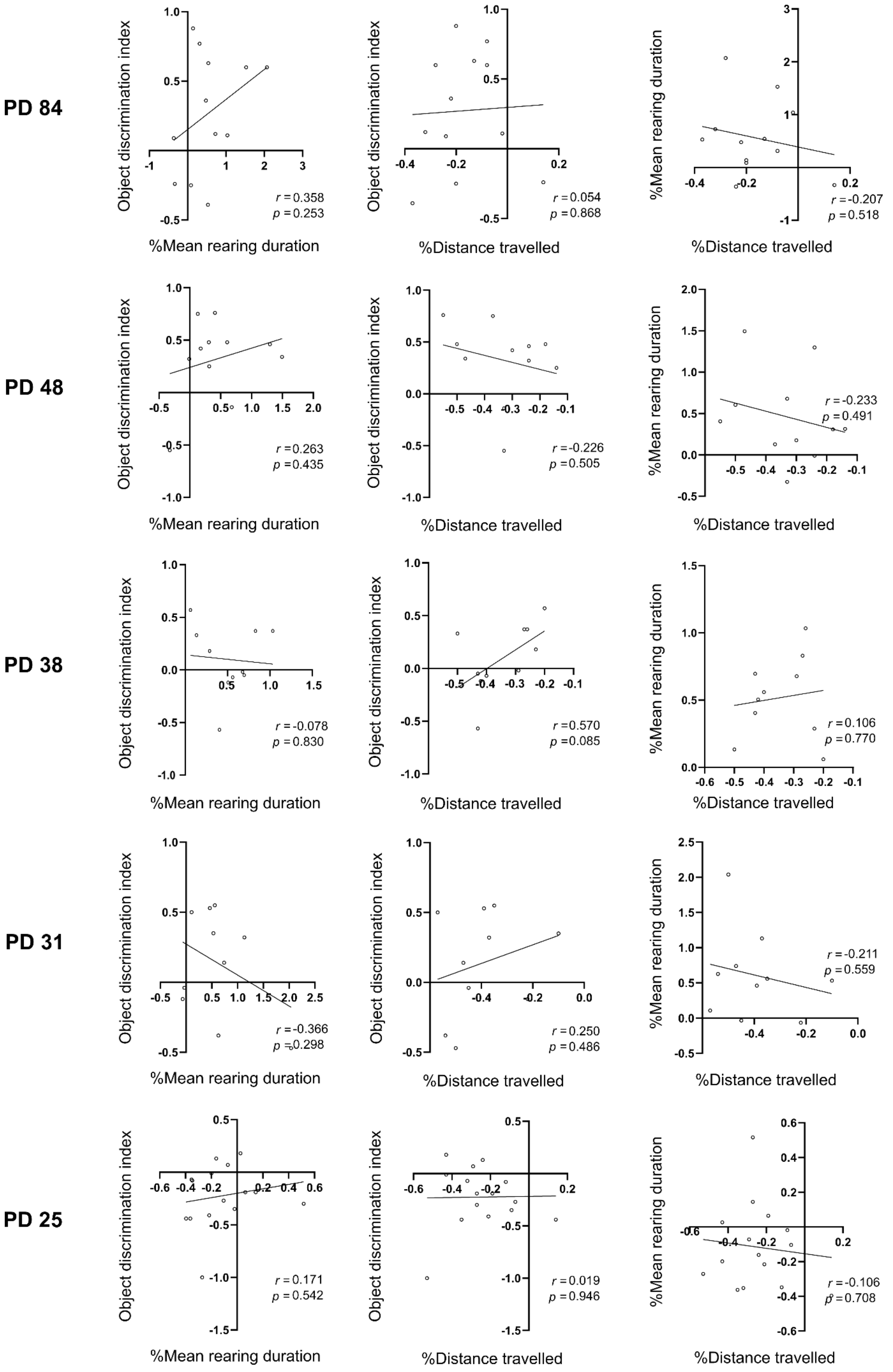
PD 84
Object discrimination index
%Mean rearing duration
r = 0.358
p = 0.253
Object discrimination index
%Distance travelled
r = 0.054
p = 0.868
%Mean rearing duration
%Distance travelled
r = -0.207
p = 0.518
PD 48
Object discrimination index
%Mean rearing duration
r = 0.263
p = 0.435
Object discrimination index
%Distance travelled
r = -0.226
p = 0.505
%Mean rearing duration
%Distance travelled
r = -0.233
p = 0.491
PD 38
Object discrimination index
%Mean rearing duration
r = -0.078
p = 0.830
Object discrimination index
%Distance travelled
r = 0.570
p = 0.085
%Mean rearing duration
%Distance travelled
r = 0.106
p = 0.770
PD 31
Object discrimination index
%Mean rearing duration
r = -0.366
p = 0.298
Object discrimination index
%Distance travelled
r = 0.250
p = 0.486
%Mean rearing duration
%Distance travelled
r = -0.211
p = 0.559
PD 25
Object discrimination index
%Mean rearing duration
r = 0.171
p = 0.542
Object discrimination index
%Distance travelled
r = 0.019
p = 0.946
%Mean rearing duration
%Distance travelled
r = -0.106
p = 0.708

**Extended Figure 5-1**. Scatter plots showing key correlations between behavioral parameters of spatial novelty detection during the retrieval phase of the OPR task. Left - Correlation between object discrimination index (ODI, y-axes) and %Mean rearing duration and (middle) %Distance travelled. To avoid complexity, the %Total rearing number variable is excluded. Right – Correlation between %Mean rearing duration (y-axis) and %Distance travelled. Pearson correlations are shown separately for each age group. *P < 0.05, **P < 0.01 and ***P < 0.001 level (uncorrected).

**Extended Data Table 1| Correlations between ODI, exploratory rearing and distance travelled for minute 3 of OPR task**

| | | | %Total rearing number | ODI | %Distance travelled |
|---|---|---|---|---|---|
| %Mean rearing duration | PD 84 | ***r*** | 0.273 | 0.358 | -0.207 |
| | | ***p*** | 0.390 | 0.253 | 0.518 |
| | PD 48 | ***r*** | -0.104 | 0.263 | -0.233 |
| | | ***p*** | 0.762 | 0.435 | 0.491 |
| | PD 38 | ***r*** | 0.470 | -0.078 | 0.106 |
| | | ***p*** | 0.171 | 0.830 | 0.770 |
| | PD 31 | ***r*** | -0.029 | -0.366 | -0.211 |
| | | ***p*** | 0.936 | 0.298 | 0.559 |
| | PD 25 | ***r*** | -0.229 | 0.171 | -0.106 |
| | | ***p*** | 0.412 | 0.542 | 0.708 |
| | Average | ***r*** | 0.085 | 0.071 | -0.132 |
| | | ***p*** | 0.566 | 0.630 | 0.107 |
| %Total rearing number | PD 84 | ***r*** | | 0.273 | 0.288 |
| | | ***p*** | | 0.391 | 0.364 |
| | PD 48 | ***r*** | | -0.465 | 0.508 |
| | | ***p*** | | 0.150 | 0.111 |
| | PD 38 | ***r*** | | 0.245 | 0.617 |
| | | ***p*** | | 0.494 | 0.057 |
| | PD 31 | ***r*** | | -0.115 | 0.820 |
| | | ***p*** | | 0.752 | **0.004** |
| | PD 25 | ***r*** | | 0.437 | 0.327 |
| | | ***p*** | | 0.103 | 0.234 |
| | Average | ***r*** | | 0.076 | 0.547 |
| | | ***p*** | | 0.684 | **0.017** |
| ODI | PD 84 | ***r*** | | | 0.054 |
| | | ***p*** | | | 0.868 |
| | PD 48 | ***r*** | | | -0.226 |
| | | ***p*** | | | 0.505 |
| | PD 38 | ***r*** | | | 0.570 |
| | | ***p*** | | | 0.085 |
| | PD 31 | ***r*** | | | 0.250 |
| | | ***p*** | | | 0.486 |
| | PD 25 | ***r*** | | | 0.019 |
| | | ***p*** | | | 0.946 |
| | Average | ***r*** | | | 0.148 |
| | | ***p*** | | | 0.366 |

**Extended Table 1.** Summary of correlations between the %Mean rearing duration, %Total rearing number, object discrimination index (ODI), and %Distance travelled for the first 3-min period of the OPR test phase. Since Kolmogorov-Smirnov tests did not support non-normality of any of the distributions, Pearson's correlation coefficients and respective p-values are indicated in bold (uncorrected). Bottom lines indicate mean correlation coefficients across age groups; p-values, here, refer to one sided t-tests (against zero) to test systematic shifts towards negative or positive correlation coefficients across age groups (mean correlations and t-tests were calculated on Fisher z-transformed coefficients).
